# Skin Cancer Excision Analysis in a Single Rural Center in Scotland’s Highlands

**DOI:** 10.7759/cureus.21954

**Published:** 2022-02-06

**Authors:** Ahmed H Helmy, Zuhdi Al-Nabulsi, Matthew Chambers, Susana Fernandez-Diaz

**Affiliations:** 1 General Surgery, Royal Glamorgan Hospital, Ynysmaerdy, GBR; 2 General Surgery, Belford Hospital, Fort William, GBR; 3 Internal Medicine, Aberdeen Royal Infirmary, Aberdeen, GBR

**Keywords:** ­skin cancer, cancer detection rate, rural areas, rural surgery, total excisional biopsy, non melanoma carcinomas, squamous cells carcinoma

## Abstract

Background & Aim

Skin cancer is the most common cancer around the world. Regional differences have been reported affecting the demographics and the prevalence of non-melanoma skin cancers; furthermore, non-melanoma skin cancers are believed to be underreported. In this study, we aim to identify and highlight any possible significant characteristics of skin cancer in our rural center in Scotland's Highlands.

Methods

This is a retrospective study analyzing and reporting cancerous skin lesions excision rates among all skin lesions excised and their characteristics in our rural center for one year. Clinical and histopathological data for patients attending our services for suspicious skin lesions excision were collected. Data included the patient’s age, gender, lesion’s diagnosis, site, size, color, borders, resection edges, recurrence, and complications. A database was created creating two cohorts: cancer and non-cancerous lesions groups, both cohorts' data was compared using student T-tests and Z-tests. P-values were considered statistically significant if < 0.5, Overall data was analyzed revealing trends and end results.

Results

From December 2019 to December 2020, 96 patients underwent skin lesions excision, 30% were cancerous. Basal cell carcinoma was the most common malignant growth standing for 76.7% of all malignant lesions excised. Squamous cell carcinoma and melanoma were found in 20% and 3.3% of patients with malignant lesions, respectively. Out of the total, 76% of cancerous lesions were in males. The most common site was head and neck (58.8%). High-risk lesions were the ones on the head and neck (P= 0.00988), in the elderly over 74.5 years (P= 0.000037), and males (P= 0.001).

Conclusion

Basal cell carcinoma was the most common malignant lesion. Elderly men with lesions on the head and neck had higher risks for cancer. Further clarification may be required with larger multi-center studies involving general practitioners, which might help identify regional variations.

## Introduction

Worldwide, skin cancer is reported as the most common cancer affecting humans [1]. In general, studies and statistics classify it into melanoma and non-melanoma. In the United Kingdom (UK), melanoma was the fifth most common cancer affecting humans in 2017 [2]. Basal cell carcinoma (BCC) is reported as the most common type of skin cancer [3] . In the UK, melanoma has higher rates of mortality. From 2015 to 2017, melanoma contributed to 1% of all cancer deaths, while non-melanoma skin cancers deaths were less than half of that occurred due to melanoma [4].

In Scotland, skin cancer incidence is rising; more than 12000 patients are diagnosed annually [5]. A 10-point guideline for urgent suspicion for referral is issued by the Scottish Referrals Guidelines Steering Group (SRGSG) to help identify and detect suspicious lesions for cancer (included in the appendices section of this article). Those points include the well-known seven points checklist [6]. The guidance suggests that suspicious lesions should be referred to dermatologist, and stresses that histopathological examination is mandatory for all excised lesions. Furthermore, excisions of lesions suspected for melanoma in primary care are discouraged [7]. This flows well with the advice from the National Institute for Health and Clinical Excellence (NICE) which states that only low-risk BCC can be excised in primary care by appropriately trained General Practitioners (GP) [3]. Our rural hospital local arrangements allow GPs to refer patients to the general surgical team for suspicions lesion excision. In this study we aim to review our practice and identify any possible significant findings.

## Materials and methods

This is a single-center retrospective analysis of skin cancer suspicious lesions excision in our rural hospital from December 2019 to December 2020. Local approval (approval number 2021/22 - 004) was obtained from Deputy Caldicott Guardian for data collection, National Health Services (NHS) Highlands. Clinical and histopathological data for patients booked for DCLA (day case under local anesthesia) were collected and analysed. Mainly patients were referred by our local GPs for suspicious skin lesions. Suspicious skin lesions were identified as skin lesions carrying a potential risk of being skin cancer. Patients were usually referred with alarming features suggestive of cancers included in the 10 points guidance from SRGSG such as change of color and size, irregular borders, inflammation, and oozing or bleeding. Excision was identified as complete lesion excision with safe borders targeting to treat potential cancer and prevent its recurrence. 

Clinical and histopathological data collected were the patient’s age, gender, Lesion’s diagnosis, site, size, color, borders, resection edges, recurrence, and complications. A Microsoft Excel database was created for two cohorts, cancer and non-cancer groups. Data in both cohorts were categorised into numerical and categorial variables. Student T-test was used to analyse numerical variables but Z-test was used to analyse categorial variables. P values in both tests were considered statistically significant if < 0.5. Overall data was analysed revealing trends and results. Kolmogorov-Smirnov test of normality was performed on overall sample numerical demographic data, such as age, and revealed that our data doesn't differ significantly from that which is normally distributed.

## Results

Ninety-six patients with average age of 58.2 years were included in our study; female to male ratio was 1.18:1 (52 females, 44 males). Cancerous lesions were found among 29 patients (30.2%). The average age of the patients with excised cancerous lesions was 74.5 years (Table 1).

**Table 1:** Demographics and Lesions Characteristics

	Non-cancer group	Cancer group	P Value
Group Population=N	67	29	-
Females	45 (67.16%)	7 (24.13%)	0.001335
Males	22 (32.83%)	22 (75.86%)	0.001335
Mean Age	52.01	74.5	0.000037
SD (Standard Deviation)	17.43	25.45	-
Multiple Lesions Analysis			
Total lesions: N=109 (SD)	70 (0.206)	39 (0.637)	-
Lesion / Patient= 1.135	1.06	1.3	0.018209
Females: Males Ratio	75:25	20:80	-
Distribution ratios			
- Head & Neck	32.39 %	58.82 %	0.00988
- Trunk - Anteriorly	16.90 %	8.82 %	0.267
- Trunk - Posteriorly	18.30 %	14.70%	0.4585
- Upper Limbs	21.12 %	17.64%	0.67448
- Lower Limbs	11.26 %	0 %	-

**Figure 1:**
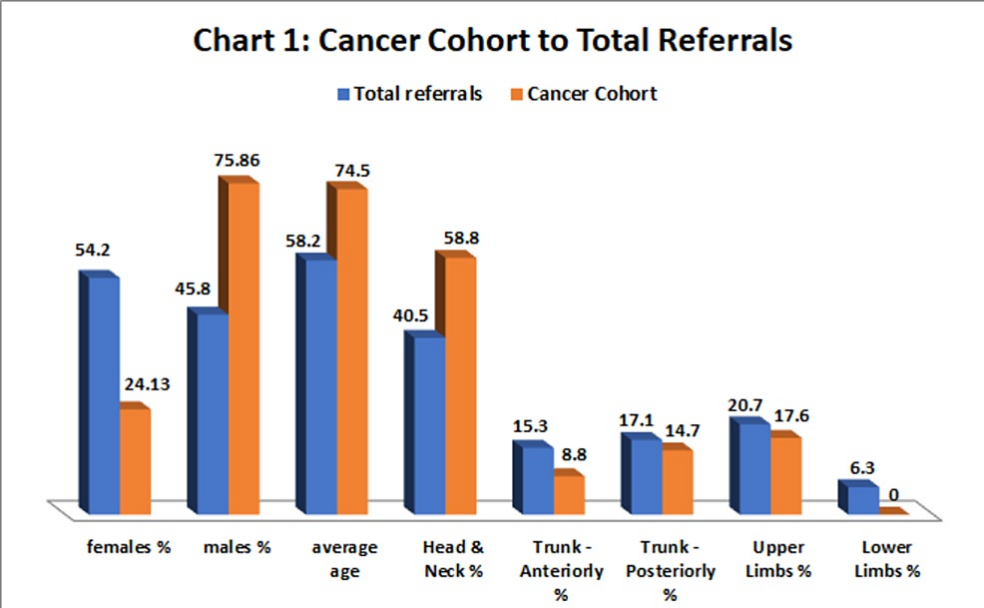
Cancer Cohort to Total Referrals

In the cancer cohort, 75.86% of cancerous lesions were found in males (Figure 1). 

Cancerous lesions identified were BCC, squamous cell carcinoma (SCC), and melanoma. BCC was the most common malignant pathology representing 76.6% of all cancerous lesions found (n= 23). SCC was found in six patients, while melanoma was found in one patient only.

Total 109 lesions were initially excised for the 96 patients. The head and neck was the most common region for all lesions excised (40.5%). Table 1 describes body regions lesion’s distributions. Thirty-four cancerous lesions out of 39 were found in 29 patients with an average ratio of 1.172 cancerous lesions per patient or 1.3 lesions per patient in the cancer cohort. Head and Neck remain the main site corresponding to 58.8 % of all cancerous skin lesions, this is followed by upper limbs (17.6%), trunk posteriorly (14.7%), trunk anteriorly (8.8%), and no cancerous lesions in the lower limbs (0%).

Margins are identified as the radial normal skin next to the lesion obtained from pathology reports which were used to obtain the analysed measurements. In the pathology reports, 86.7% of the cancerous lesions had a documentation of margins. Average resected margins obtained was 1.5 mm, this ranged from 0 mm (no margins) to 4 mm. Out of the total, 13% (n =4) of the cancer cohort appeared to have non-sufficient margins, 50% (n=2) of those had their lesions on the face, one patient had malignant melanoma and another had BCC.

Patient’s demographics data and lesions site distribution analysed showed the following characteristics carrying a significant risk for cancer; lesions found on the head and neck (P= 0.00988), in elderly patients over 74.5 years old (P= 0.000037) and in males (P= 0.000062), the more lesions present in single patients the more risk of being cancerous.

## Discussion

Non-melanoma skin cancer is considered the most common prevalent cancer in Scotland; in 2016 its incidence was more than 25% of all cancers diagnosed at 11,677 cases. Nearly 75% of Non-melanoma skin cancer is found in people over 65 years old and it is diagnosed in males more than females 1.4:1 [7,8]. In our review, the average age of patients diagnosed with cancerous lesions was 74.5 years; 80% of them were aged 65 years or older. Only 23.3% of cancerous lesions were found in females although they represented 54.16% of all patients presenting for suspicious skin lesions, this flows with the results reported by Haw et al. in 2014 where more cancerous skin lesions were found in males to females in South East Scotland, 58% to 42% respectively [9].

**Figure 2:**
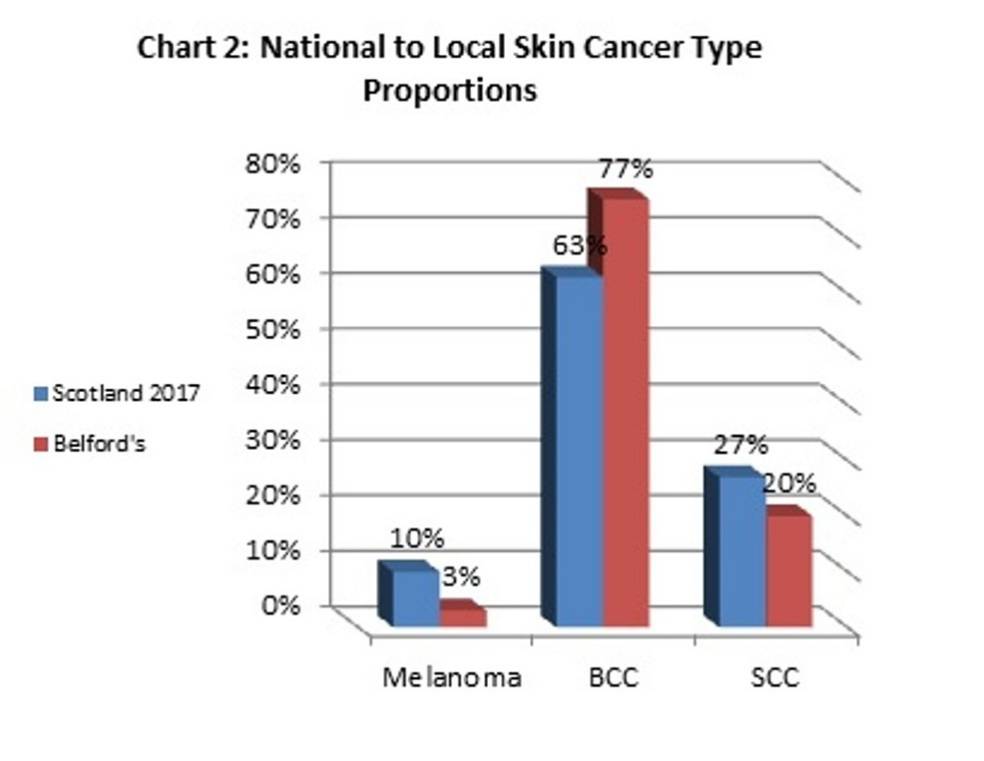
National to Local Skin Cancer Type Proportions

In Scotland, available data breakdown in 2017 showed that BCC counted for around 70% of all skin cancers diagnosed [8]. This flows with our results where just over three-quarters of cancerous lesions diagnosed were BCC (Figure 2). BCC is associated with exposure to ultraviolet radiation, hence it is diagnosed more on sun-exposed body parts such as the head and neck, which are reported to be the most common site of BCC (80%). This percentage is greater than what we found in our small sample; where head and neck counted for nearly 60% for both BCC lesions patient’s cohort and total cancer cohort [10].

BCC usually has limited local spread, recurrence rates can increase due to several factors such as larger lesions (diameter > 2 cm), incomplete excision, or associated with aggressive histological growth [10]. Non-melanoma skin cancers are reported to have low mortality rates. In 2016, in Scotland among the 100,000 patients who have been diagnosed once with skin cancer, less than one in 1000 deaths were reported [8].

In 2020 an interesting large multi-center retrospective study on over 4700 participants reported nearly 22% of patients having total body surface examination were found to have incidental non-expected cancerous skin lesions. This study achieved a higher detection rate compared to a lesion-centered investigation where 10.9% of suspicious lesions were found to be malignant. This large study might reassure our smaller sample and referral pathways being efficient with the 30% detection rate we achieved. Furthermore, it supports and reflects the efforts needed to detect cancerous skin lesions [11].

We obtained the data of the final margin measurements pathology reports as most operative notes did not include it. Furthermore, a discrepancy was noted upon comparing both measurements when available. This is believed mainly due to the shrinkage of samples. It has been reported that margins on pathology reports do not resemble the one excised at the time of procedures as shrinkage is believed to occur immediately as soon as the sample is excised [12]. 

SRGSG states in their guidance that suspicious skin lesions should be referred to dermatologists where available; however, this indeed would be not applicable in our rural settings. Hence, our local arrangements allow GPs to refer suspicious skin lesions to the general surgical team who will proceed with excision and arrange the follow-up needed, including referral to dermatologist or discussion with skin cancer multi-discipline team (MDT) if needed [6]. It is believed that our local GPs rarely perform such procedures with respect to the guidance from SRGSG which contraindicate NICE advice which states that GPs can resect small BCC lesions if appropriately trained [2]. In their 2014 study analyzing skin cancer excision in primary and secondary care in several Scottish regions, Haw et al. found that GPs have lower rates of complete cancerous skin lesion excision [9].

Our study is mainly limited by the small sample obtained, which was mainly due to our relatively small rural settings and lack of panoramic view of data from previous years. Such a small sample is not strong enough to judge why skin melanoma, despite being the fifth most common cancer in the UK, was detected only in 1% of our study populations. This could reflect that melanoma is not common in the region Scotland Highlands West, or underdiagnosed. Further clarification may be required with larger multi-center studies including GPs auditing cancerous skin lesions and their characteristics which might help identify regional variations.

## Conclusions

Although most of the lesions excised appeared to be non-cancerous, our detection rate was efficient and more than what is usually expected and reported. Basal cell carcinoma was the most common malignant lesion, whereas melanoma was barely prevalent. Elderly men with lesions on the head and neck appeared to carry higher risks for cancer. Dermatologists were barely involved, this contradicts the guidance from SRGSG, hence establishing a frequent dermatology clinic in our rural hospital or providing further training might help increase the safety index. 

## Appendices

**Table 2:** Urgent suspicion of cancer referral

Urgent suspicion of cancer referral
Lesions on any part of the body which have one or more of the following features:
Change in color, size, or shape in an existing mole
Moles with Asymmetry, Border irregularity, Color irregularity, Diameter increasing or> 6mm
New growing nodule with or without pigment
Persistent (more than four weeks) ulceration, bleeding, or oozing
Persistent (more than four weeks) surrounding inflammation or altered sensation
New or changing pigmented line in a nail or unexplained cession in a nail
Slow growing, non-healing or keratinizing lesions with induration (thickened base)
Any melanoma or invasive SCC or a high-risk BCC diagnosed from a biopsy
Any unexplained skin lesion in an immune-suppressed patient
BCC invading potentially dangerous areas, for example, periocular auditory meatus or any major vessel or nerve
